# The association of Carbohydrate Quality Index with cardiovascular disease risk factors among women with overweight and obesity: A cross-sectional study

**DOI:** 10.3389/fnut.2022.987190

**Published:** 2022-09-08

**Authors:** Darya Khosravinia, Farideh Shiraseb, Atieh Mirzababaei, Elnaz Daneshzad, Shahin Jamili, Cain C. T. Clark, Khadijeh Mirzaei

**Affiliations:** ^1^Department of Nutrition, Science and Research Branch, Islamic Azad University, Tehran, Iran; ^2^Department of Community Nutrition, School of Nutritional Sciences and Dietetics, Tehran University of Medical Sciences, Tehran, Iran; ^3^Non-Communicable Diseases Research Center, Alborz University of Medical Sciences, Karaj, Iran; ^4^Department of Surgery, Shahid Beheshti, Fellowship of Minimally Invasive Surgery, Tehran, Iran; ^5^Centre for Intelligent Healthcare, Coventry University, Coventry, United Kingdom; ^6^Food Microbiology Research Center, Tehran University of Medical Sciences, Tehran, Iran

**Keywords:** cardiovascular disease risk factors, anthropometric measures, body composition, obesity, carbohydrate quality index

## Abstract

**Purpose:**

Diet is one of the most important factors influencing cardiovascular disease (CVD). The negative relationship between carbohydrate intake with lipid profiles and body weight has been previously investigated. However, this is the first study seeking to assess the association of carbohydrate quality index (CQI) with CVD risk factors.

**Methods:**

This cross-sectional study was conducted on 291 Iranian overweight and obese women, with a body mass index (BMI) ranging between 25 and 40 kg/m^2^, and aged 18–48 years. CQI scores were calculated by using a validated 168-item semi-quantitative food frequency questionnaire (FFQ). Biochemical and anthropometric measures were assessed using standard methods, and bioelectrical impedance was used to measure body composition.

**Results:**

We observed that fruits (*P* < 0.001), vegetables (*P* < 0.001), and protein (*P* = 0.002) intake were higher in participants with a higher score of the CQI. When we adjusted for potential confounders, we observed that the CQI was negatively related to systolic blood pressure (SBP) (β = −6.10; 95% CI = −10.11, −2.10; *P* = 0.003) and DBP (β = −3.11; 95% CI = −6.15, −0.08; *P* = 0.04). Also, greater adherence to a high CQI dietary pattern, compared to the reference group, was negatively related to HOMA-IR (β = −0.53; 95% CI = −0.94, −0.12) (P for trend = 0.01), WC (β = −3.18; 95% CI = −6.26, −0.10) (*P* for trend = 0.04), BMI (β = −1.21; 95% CI = −2.50, 0.07) (*P* for trend = 0.06), and BF (β = −2.06; 95% CI = −3.82, −0.30) (*P* for trend = 0.02).

**Conclusion:**

In line with previous studies, the CQI was inversely associated with blood pressure, WC, BMI, and BF. Further prospective and clinical trial studies are suggested to confirm these data.

## Introduction

Obesity is one of the most profound medical problems in the world that increases the risk of other chronic diseases, such as cardiovascular disease, cancers, and diabetes (1). According to the World Health Organization (WHO), more than 1.9 billion adults, over 18 years, were overweight in 2016, and more than 650 million of them were obese (2, 3). It is estimated that, by 2030, 2.5 billion people will be overweight or obese (4, 5). Women appear to be more affected by the obesity epidemic than men, where this difference may be related to nutrition, lifestyle, behavior, sexual, and environmental differences (6, 7). Moreover, a higher risk of cardiovascular disease (CVD) in women has been observed, especially in women with obesity or overweight (8–10). Factors influencing the incidence and prevalence of obesity include genetic and environmental factors, such as lifestyle and eating habits (11, 12). Further diet is one of the most important factors influencing chronic inflammatory conditions (13).

Special diets have been suggested for the maintenance of optimal body weight. However, their results are controversial (14). Some studies have investigated the role of macronutrients, especially carbohydrates as the main source of energy by Iranians, in the development of obesity (14). Accordingly, low carbohydrate diets (LCD) were reported as a common weight-loss strategy (15). Interestingly, a systematic review showed no association between high carbohydrate intake with the risk of obesity (16). The results of one trial revealed that the LCD may reduce body mass and fat content (17); however, they did not consider the calorie intake and carbohydrate quality (18). The quality of dietary carbohydrates may be more important than their quantity in reducing the risk of CVD (19, 20). Also, one factor alone is insufficient to predict the association between carbohydrate intake with obesity risk, and so, carbohydrate quality should be determined by considering several important factors simultaneously (18). Thus, Carbohydrate Quality Index (CQI) was defined in which fiber intake, glycaemic index (GI), whole grains/total grains ratio, and solid carbohydrate/total carbohydrate ratio are calculated (21). In a cohort study, a negative relationship between CQI with obesity was shown (22). Another study proposed that CQI components, such as GI, significantly affected abdominal obesity (23). Moreover, a previous study concluded that fiber intake elicited weight-loss and body fat (BF) loss, compared to refined grains (24). Also, a low GI diet may be associated with a decrease in body fat mass (BFM) (25, 26).

The association between obesity with CVD in men was reportedly related to high blood pressure and cholesterol (27, 28). Thus, controlling these two factors can be effective in reducing the risk of CVD. The results of prospective cohort studies have shown that each 5 kg/m^2^ higher body mass index (BMI) is associated with a 27% higher risk of chronic heart disease (29). A cohort study showed an increment in high-density lipoprotein (HDL), low-density lipoprotein (LDL), triglyceride (TG), and total cholesterol (TC) in obese participants (30). Moreover, a meta-analysis demonstrated LCD can lead to a decrease in body weight, waist circumference (WC), BMI, TG, and blood pressure (31). A positive relationship between CQI with HDL levels was observed in a study (32). In a Mediterranean cohort study, an inverse relationship was observed between better CQI with the incidence of CVD (33). In another study, the CQI had a positive relationship with HDL levels, and a negative relationship with systolic blood pressure (SBP), diastolic blood pressure (DBP), TG, and WC (34).

Several mechanisms have been proposed for these relationships, including the association of high GI foods with hyperinsulinemia, increased fat storage, and reduced blood glucose fluctuations, which leads to increased appetite and food intake (35–37). Fiber intake decreases appetite through slowing stomach emptying and hormone signaling, and decreases postprandial insulin that increases lipid oxidation (38–42), whilst high liquid carbohydrate intake increases appetite and postprandial blood glucose and decreases insulin sensitivity (43, 44), and whole grains can reduce the digestion and absorption of starch and appetite (38, 45).

To our knowledge, there is no previous study investigating the relationship between CQI with CVD risk factors in Iranian women. Therefore, due to the high prevalence of CVD and its importance, we intended to determine the relationship between CQI and CVD risk factors among women with overweight and obesity.

## Materials and methods

### Study population

This cross-sectional study was conducted on 291 overweight and obese women, aged 18–48 y, who were recruited from health centers in Tehran, Iran. The BMI of women ranged between 25 and 40 kg/m^2^. Exclusion criteria included: history of any chronic diseases such as diabetes mellitus, hypertension, CVDs, liver or kidney diseases, taking all types of medicine including birth control pills, smoking, intake of alcohol, pregnancy, lactating women, post-menopause, body weight changes in the last year, weight-loss diets or an arbitrary special dietary regimen, and chronic disease that affected their diet. All participants signed a consent form before starting this study. Our study was approved by the local ethics committee of the Tehran University of Medical Sciences. The approval number was IR.TUMS.MEDICINE.REC.1400.182.

### Assessment of dietary intake and CQI calculation

A reliable semi-quantitative food frequency questionnaire (FFQ) was used for obtaining the usual dietary intake of participants during the past year. This FFQ included 168 items, where standard portion size, and food frequency categories (daily, weekly, monthly, and yearly) for each food which was converted to grams per day using household measurements (46). This FFQ was collected with a face-to-face interview by a trained interviewer, and Nutritionist-4 software was used to analyze the data.

The area below the glycemic response curve for each participant based on the reference food was shown as a percentage of the average area under the curve, after each food. Food GI for all participants was calculated by mean of these values. White bread was used as a reference food. GI values were multiplied by 0.71 to convert the glucose scale (i.e., GI glucose = 100) (47). Total GI was estimated using the following formula: (GI × available carbohydrates)/total available carbohydrates. To calculate available carbohydrates, fiber was deducted from total carbohydrates, which were derived from the United States Department of Agriculture food composition databases (48).

CQI was computed by summing the following four criteria: dietary fiber intake (g per day), GI, the ratio of whole grains to total grains, and the ratio of solid carbohydrate to total carbohydrate. Total grains include whole grains, refined grains, and their products. For each of these four components, a score of 1–5 was considered. Finally, CQI is obtained and ranges from 4 to 20; participants were subsequently categorized into tertiles (28).

### Anthropometric measurements and body composition analysis

Weight was measured on a digital scale, where participants were weighed with minimal clothing and without shoes, to the nearest 100 g. Participants' height was measured, without shoes, to the nearest 0.5 cm. WC and hip circumference (HC) were measured to the nearest 0.5 cm, according to standard procedures. Subsequently, waist-to-hip ratio (WHR) and BMI were calculated according to standard formulae. According to WHO guidelines, overweight and obesity were defined as 25 ≤ BMI ≤ 29.9 kg/m^2^ and BMI ≥ 30 kg/m^2^, respectively. Neck circumference (NC) was measured by the use of non-stretchable plastic tape, to the closest 1 mm, just underneath the laryngeal prominence perpendicular to the long axis of the neck with the head placed within the Frankfort horizontal aircraft (49). The body composition of participants was measured by a Body Composition Analyzer BC-418MA- In Body (United Kingdom), according to manufacturer guidelines. Participants were asked not to exercise, not to use any electrical devices, and not to consume excessive fluid or food before measuring the body composition, to prevent any discrepancies in the measured values.

### Biochemical assessment

After 10–12 h of fasting at night, a blood sample was drawn and serum was collected into tubes containing 0.1% Ethylenediaminetetraacetic acid (EDTA). Then, they were centrifuged for 10 min at 3,000 rpm, aliquoted into 1 ml tubes, and stored at −70°C until analysis. Sample analysis was performed by using an autoanalyzer (Selectra 2; Vital Scientific, Spankeren, Netherlands). The FBS was measured by using the GOD/PAP (glucose oxidase phenol 4-Aminoantipyrine Peroxidase) method. The serum levels of HDL and LDL were determined by turbidimetry on a Roche Hitachi analyzer (Roche, Germany). The blood levels of TG and TC were determined by using an enzymatic technique and commercially available Pars Azmoon, Iran kits. Also, a high-sensitivity immunoturbidimetric assay (Hitachi 902 analyzer; Hitachi Ltd, Tokyo, Japan) was used to measure serum high-sensitivity C-reactive protein (hs-CRP). Furthermore, the homeostasis model assessment method was used to determine insulin resistance *via* the HOMA-IR formula as follows: fasting serum insulin (mlU/L) × FBS (mmol/L)/22.5 (50). HOMA-IR cut-off values > 2.63 are considered as the presence of insulin resistance (51).

### Blood pressure assessment

The blood pressure of participants was measured by a standard mercury sphygmomanometer (ALPK2 k2-232; Japan), while the participants were sitting for 10–15 min, before performing two consecutive measurements. Two measurements were performed at 1 min intervals and the average was considered.

### Physical activity

Participants' physical activity was assessed by the short form of the international physical activity questionnaire (IPAQ), according to the frequency and time of common activities of daily life over the past year. Physical activity levels were expressed as metabolic equivalent minutes per week (MET-minutes/week) (52) and were divided into categories as follows: very low (<600 MET-min/week), low (600–3,000 MET-min/week), moderate, and high (>3,000 MET-min/week) (53).

### Assessment of other variables

A demographic questionnaire was used to collect information about age, marital status, education, occupation, economic status, and supplementation.

### Statistical analysis

The minimum sample size was 152 people through the following formula and with a design effect of 1.5: *n* = (([(Z_1−α_ + Z_1−β_) × √1 – r^2^]/r)^2^ + 2), which α = 0.05, β = 0.95, *r* = 0.3 (54). Quantitative variables were described as mean and standard deviation (SD) and categorical variables were described as numbers and percentages. The Kolmogorov-Smirnov test was used to check the distribution of data (*P* > 0.05, indication normal distribution). All statistical analyses were performed by SPSS software version 26, and *P* < 0.05 was considered to be statistically significant, and *P* = 0.05–0.07 was considered marginally significant. By use of the NOVA score, participants were categorized into tertiles. Individuals in tertile 1 were 103 (35.4%) with a threshold of <10, in tertile 2 were 99 (34%) with a threshold of 10–13, and 89 (30.6%) for tertile 3 with a threshold of >13. To compare the mean difference of quantitative variables and percent of categorical variables across NOVA tertiles, one-way analysis of variance (ANOVA) and chi-square (χ^2^) tests were performed, respectively. Analysis of covariance (ANCOVA), controlling for potential confounders (age, BMI, energy intake, and physical activity) and considering BMI as a collinear variable for anthropometric measures and body composition variables, was also conducted. Bonferroni *post-hoc* testing was done to identify the exact location of significant mean differences among tertiles, if necessary. Linear regression was conducted to determine the association between CQI and CVD risk factors. Model 1 was adjusted for age, BMI, energy intake, and physical activity, and Tertile 1 was considered as the exposure reference group. The results were reported as β, with a 95% confidence interval (95% CI).

## Results

### Study population

This cross-sectional study was conducted on 291 overweight and obese women, of whom, 72.2% were married, 97.9% were employed, 48.8% had a college education, and 23% had a poor economic level. The mean (SD) of age, weight, BMI, and WC of participants was 36.51 (8.51) years, 80.71 (12.22) kg, 31.05 (4.32) kg/m^2^, and 98.96 (10.04) cm, respectively. Also, the mean (SD) CQI of participants was 11.83 (3.12). Other main demographic quantitative and qualitative variables are shown in Table 1.

**Table 1:** General characteristics of the study participants (*n* = 291).

**Variables**	**Mean**	**SD**	**Minimum**	**Maximum**
**Demographics**				
Age (years)	36.519	8.511	17	56
PA (MET-minutes/week)	998.39	1,089.26	10	7,296
**Blood parameters**				
FBS (mg/dl)	87.49	9.62	67	137
TC (mg/dl)	185.15	36.25	104	344
TG (mg/dl)	118.30	59.78	37	328
HDL (mg/dl)	46.80	10.85	18	87
LDL (mg/dl)	95.03	24.19	34	156
hs-CRP (mg/L)	4.31	4.65	0.00	22.73
**Blood pressure**				
SBP (mmHg)	111.65	13.75	76	159
DBP (mmHg)	77.77	9.62	51	111
**Anthropometric parameters**				
Weight (kg)	80.71	12.22	59.50	136.60
Height (cm)	161.28	5.93	142	179
BMI (kg/m^2^)	31.05	4.32	24.20	49.60
WC (cm)	98.96	10.04	80.10	136
WHR	1.24	5.34	0.81	0.92
HC (cm)	114.14	9.75	100	160
NC (cm)	37.56	7.39	31	134.5
**Body composition**				
FFM (kg)	46.78	5.58	35.30	63
BFM (kg)	34.01	8.67	19.40	74.20
BF (%)	41.51	5.53	15	54.30
VFA (cm^2^)	168.28	104.83	20	1,817
VFL (cm)	16.66	14.17	7	208.4
FFMI	18.40	7.78	14.6	147.8
FMI	13.15	3.39	6.9	26.9
TF (kg)	16.56	3.70	9.7	30.2
TF (%)	313.74	70.02	177.8	536.6
**CQI components**				
CQI	11.83	3.12	4	19
Fiber (g/day)	41.00	14.35	8.61	87.89
Glycaemic index	56.75	6.13	40.50	67.69
Solid CHO (g/day)/ total CHO (g/day)	0.71	0.21	−0.75	1
Whole grain (g/day) to total grain (g/day)	0.02	0.03	0.00	0.31
HOMA-IR index	3.34	1.28	1.29	9.19
**Categorical variables**	**Status**	***N***	**%**	
Marriage status	Single	87	26.8	
Occupation	Unemployed	2	0.7	
Education	Illiterate	3	1	
	Under diploma	36	12.4	
	Diploma	107	36.8	
	Bachelor and higher	142	48.8	
Level of economic status	Poor	67	23	
	Moderate	138	47.4	
	Good	72	224.7	
Supplementation	Yes	134	46	

BF, body fat percentage; BFM, body fat mass; BMI, body mass index; CHO, carbohydrates; CQI, carbohydrate quality index; DBP, diastolic blood pressure; FBS, fasting blood sugar; FFM, fat-free mass; FFMI, fat-free mass index; FMI, fat mass index; HDL, high-density lipoprotein; HOMA-IR, homeostatic model assessment for insulin resistance; hs-CRP, high-sensitivity C-reactive protein; LDL, low-density lipoprotein; N, number; NC, neck circumference; PA, physical activity; SBP, systolic blood pressure; SD, standard deviation; TC, total cholesterol; TG, triglyceride; TF, trunk fat; VFA, visceral fat area; VFL, visceral fat level; WC, waist circumference; WHR, waist-hip ratio.

Quantitative variables were obtained from one-way ANOVA and presented as mean ± SD, and qualitative variables were obtained from the Chi-Square test and presented as frequency and percentage.

### General characteristics of study participants among tertiles of the CQI

Based on Table 2, the participants with a higher score of CQI were older (*P* = 0.002). Although the participants with a higher score of CQI had a lower mean weight, there was no significant difference between the anthropometric measures and other general characteristics of participants across tertiles of CQI (*P* > 0.05).

**Table 2:** General characteristics of study participants among tertiles of the CQI (*n* = 291).

**Characteristics**		**Tertiles of CQI**			*P*-value^*^	*P*-value^**^
		**T1**	**T2**	**T3**		
		***n* = 103**	***n* = 99**	***n* = 89**		
		**Mean ±SD**	**Mean ±SD**	**Mean ±SD**		
		** <10**	**10–13**	**>13**		
**Demographics**						
Age (years)^a^		34.51 ± 9.14	37.22 ± 8.68	38.06 ± 7.09	**0.009**	**0.002**
PA (MET-minutes/week)		884.59 ± 731.34	938.46 ± 1,024.15	1,160.41 ± 1,407.53	0.24	0.07
**Anthropometric parameters**						
Weight (kg)		82.33 ± 14.09	80.28 ± 11.94	79.31 ± 9.90	0.21	0.47
Height (cm)		161.41 ± 5.62	160.70 ± 5.93	161.78 ± 6.26	0.44	0.66
HC (cm)		115.33 ± 12.02	113.04 ± 8.41	113.83 ± 7.62	0.43	0.99
Insulin (mlU/ml)		1.20 ± 0.25	1.21 ± 0.22	1.23 ± 0.20	0.76	0.54
**Categorical variables**		***N*** **(%)**				
Marriage status	Single	23 (29.5)	30 (38.5)	25 (32.1)	0.41	0.29
	Married	79 (37.6)	68 (32.4)	63 (30.0)		
Occupation	Unemployed	1 (50.0)	0 (0.0)	1 (50.0)	0.75	0.58
	Employed	101 (35.4)	98 (34.4)	86 (30.2)		
Education status	Illiterate	2 (66.7)	1 (33.3)	0 (0.0)	0.81	0.66
	Under diploma	15 (41.7)	13 (36.1)	8 (22.2)		
	Diploma	36 (33.6)	36 (33.6)	35 (32.7)		
	Bachelor and higher	49 (34.5)	48 (33.8)	45 (31.7)		
Level of economic status	Poor	21 (31.3)	22 (32.8)	24 (35.8)	0.27	0.91
	Moderate	49 (35.5)	54 (39.1)	35 (25.4)		
	Good	28 (38.9)	19 (26.4)	25 (34.7)		
Supplementation	Yes	44 (32.8)	46 (34.3)	44 (32.8)	0.80	0.99
	No	37 (37.0)	32 (32.0)	31 (31.0)		

HC, hip circumference; N, number; PA, physical activity; SD, standard deviation; T, tertile.

Participants were divided into categories called tertiles.

* The *P*-values were obtained by the use of ANOVA or the Chi-Square test.

** The *P*-values were obtained by the use of ANCOVA after adjustment for age, BMI, energy intake, and physical activity (MET-minutes/week). BMI is considered a collinear for anthropometric measurements.

*P* < 0.05 was considered statistically significant and *P* = 0.05–0.07 was considered marginally significant.

Quantitative variables were obtained from one-way ANOVA and presented as mean ± SD, and categorical variables were obtained from the Chi-Square test and presented as frequency and percentage.

Carbohydrate quality index includes total fiber, glycemic index, whole grains to total grains ratio, and solid carbohydrate to total carbohydrate ratio.

a Significant difference with Bonferroni analysis was seen between T1 and T3.

Bold values indicate significant and marginally significant *p*-values.

### Dietary intake of study participants among tertiles of the CQI

As shown in Table 3, energy (*P* = 0.01), protein (*P* = 0.002), and carbohydrate (*P* = 0.001) intake were higher in participants with a higher score of the CQI and total fat intake was lower after controlling for potential confounder (energy intake) (*P* < 0.001). participants with a higher score of CQI had a significantly lower intake of saturated fatty acids (SFA), and a higher intake of vegetables, legumes, whole grains, potassium, calcium, phosphorus, iron, magnesium, copper, vitamin K, vitamin B1, vitamin B2, vitamin B6, vitamin B8, and vitamin B9 consumption after adjusting for energy intake (*P* < 0.001). In addition, these differences were significant for fruits (*P* = 0.003), nuts (*P* = 0.06), total fiber (*P* = 0.01), zinc (*P* = 0.007), vitamin A (*P* = 0.001), vitamin C (*P* = 0.03), vitamin B3 (*P* = 0.003), and pantothenic acid (*P* = 0.01) intake. Monounsaturated fatty acids (MUFA) (*P* < 0.001), polyunsaturated fatty acids (PUFA) (*P* = 0.06), and linoleic acid (*P* = 0.04) intake were lower in participants with a higher score of CQI after energy intake controlling. However, there was no significant mean difference between the other dietary components intake of participants across tertiles of the CQI (*P* > 0.05).

**Table 3:** Dietary intake of study participants among tertiles of the CQI (*n* = 291).

**Dietary intake**	**Tertiles of CQI**			*P*-value^*^	*P*-value^**^
	**T1**	**T2**	**T3**		
	***n* = 103**	***n* = 99**	***n* = 89**		
	**Mean ±SD**	**Mean ±SD**	**Mean ±SD**		
	** <10**	**10–13**	**>13**		
**CQI components**					
Fiber intake (gr/day)^a,b,c^	31.55 ± 8.71	42.26 ± 13.79	50.54 ± 13.43	**<0.001**	**<0.001**
Glycemic index^a,b,c^	59.61 ± 5.30	56.40 ± 5.82	53.82 ± 5.95	**<0.001**	**<0.001**
Solid CHO (g/day)/total CHO (g/day)^a,b,c^	0.59 ± 0.28	0.74 ± 0.13	0.81 ± 0.09	**<0.001**	**<0.001**
Whole grain (g/day)/ total grain (g/day)^b,c^	0.01 ± 0.01	0.02 ± 0.02	0.04 ± 0.04	**<0.001**	**<0.001**
**Food group components**					
Energy (Kcal/day)	2,601.05 ± 751.49	2,472.36 ± 805.38	2,783.37 ± 658.46	**0.01**	–
Fruits (g/day)^b^	457.51 ± 292.92	502.25 ± 326.31	641.16 ± 373.24	**<0.001**	**0.003**
Vegetables (g/day)^b,c^	347.97 ± 211.43	402.94 ± 261.79	566.71 ± 269.61	**<0.001**	**<0.001**
Nuts (g/day)^b^	13.08 ± 14.25	11.78 ± 11.38	18.72 ± 21.32	**0.008**	**0.06**
Legumes (g/day)^b,c^	38.41 ± 25.69	48.46 ± 36.48	73.91 ± 51.51	**<0.001**	**<0.001**
Dairy (g/day)	366.78 ± 245.37	355.98 ± 203.60	446.13 ± 280.98	**0.02**	0.19
Eggs (g/day)	19.64 ± 14.94	20.98 ± 12.56	24.82 ± 14.56	**0.03**	0.10
Fish and seafood (g/day)	10.46 ± 10.06	11.91 ± 12.70	11.94 ± 13.71	0.61	0.49
Red meat (g/day)	20.95 ± 16.74	21.17 ± 20.12	22.42 ± 18.78	0.84	0.72
Whole grains (g/day)^b,c^	3.76 ± 7.15	5.73 ± 9.51	14.07 ± 11.55	**<0.001**	**<0.001**
Refined grains (g/day)	437.70 ± 190.25	420.82 ± 234.69	438.96 ± 237.00	0.81	0.48
Total fiber (g/day)	35.43 ± 13.61	43.93 ± 18.53	57.55 ± 17.28	**<0.001**	**0.01**
Caffeine (g/day)	156.80 ± 105.56	159.85 ± 211.45	135.21 ± 111.61	0.48	0.24
Tea and coffee (g/day)	746.58 ± 519.84	781.56 ± 1057.51	687.49 ± 575.38	0.69	0.40
**Macronutrients**					
Protein (g/day)^a,b^	83.64 ± 26.88	85.40 ± 32.45	96.89 ± 23.45	**0.002**	**0.004**
Carbohydrate (g/day)^b,c^	358.79 ± 121.63	349.61 ± 122.27	411.70 ± 107.60	**0.001**	**<0.001**
Total fat (g/day)^b^	99.72 ± 34.58	89.39 ± 35.10	93.70 ± 28.85	0.08	**<0.001**
**Fatty acid subtypes**					
Saturated fatty acids (g/day)^b^	30.04 ± 10.99	26.12 ± 11.53	27.69 ± 10.47	**0.04**	**<0.001**
Cholesterol (g/day)	252.62 ± 105.68	244.20 ± 109.99	261.20 ± 97.82	0.54	0.82
MUFA (g/day)^b,c^	33.28 ± 12.91	30.13 ± 12.10	30.27 ± 9.93	0.10	**<0.001**
PUFA (g/day)^b^	21.16 ± 11.03	19.26 ± 8.53	19.65 ± 6.98	0.29	**0.06**
Linoleic acid (g/day)^b^	18.48 ± 10.43	16.66 ± 8.12	16.75 ± 6.62	0.24	**0.04**
Linolenic acid (g/day)	1.30 ± 0.79	1.09 ± 0.55	1.31 ± 0.62	**0.04**	0.22
EPA (g/day)	0.02 ± 0.03	0.03 ± 0.03	0.03 ± 0.04	0.42	0.47
DHA (g/day)	0.09 ± 0.10	0.10 ± 0.10	0.11 ± 0.13	0.51	0.54
Trans fatty acids (g/day)	0.000 ± 0.003	0.000 ± 0.001	0.001 ± 0.002	0.56	0.69
**Micronutrients**					
Sodium (mg/day)	4,226.48 ± 1,586.18	4,174.70 ± 1,460.59	4,321.61 ± 1,173.02	0.77	0.29
Potassium (mg/day)^a,b,c^	3,939.40 ± 1,505.00	4,080.78 ± 1,480.07	4,987.73 ± 1,475.67	**<0.001**	**<0.001**
Calcium (mg/day)^b,c^	1,077.20 ± 417.72	1,092.70 ± 368.99	1,325.94 ± 415.61	**<0.001**	**<0.001**
Phosphorus (mg/day)^a,b^	1,533.38 ± 507.09	1,562.34 ± 523.42	1,814.77 ± 471.73	**<0.001**	**<0.001**
Iron (mg/day)^a,b,c^	16.88 ± 5.48	17.89 ± 6.39	21.36 ± 4.90	**<0.001**	**<0.001**
Zinc (mg/day)^b^	12.32 ± 4.17	12.21 ± 4.33	14.18 ± 3.75	**0.001**	**0.007**
Selenium (μg/day)^a^	113.03 ± 41.36	118.05 ± 45.96	128.79 ± 38.48	**0.03**	**0.03**
Magnesium (mg/day)^a,b,c^	421.51 ± 143.40	433.00 ± 148.64	523.26 ± 128.15	**<0.001**	**<0.001**
Copper (mg/day)^a,b,c^	1.76 ± 0.59	1.92 ± 0.82	2.30 ± 0.57	**<0.001**	**<0.001**
Manganese (mg/day)	6.67 ± 2.45	6.91 ± 3.35	7.62 ± 2.44	**0.05**	0.17
Chromium (mg/day)	0.10 ± 0.08	0.10 ± 0.08	0.12 ± 0.08	0.22	0.48
Vitamin A (IU/day)^b,c^	694.11 ± 388.69	710.08 ± 378.48	924.52 ± 420.04	**<0.001**	**0.001**
Vitamin D (μg/day)	1.73 ± 1.67	1.90 ± 1.52	2.25 ± 1.60	0.08	0.23
Vitamin E (mg/day)^**c**^	17.64 ± 9.54	17.82 ± 10.48	16.31 ± 7.38	0.48	**0.06**
Vitamin K (μg/day)^b,c^	164.56 ± 107.08	194.32 ± 159.80	284.28 ± 265.64	**<0.001**	**<0.001**
Vitamin C (mg/day)^b^	171.90 ± 105.43	191.20 ± 149.65	225.51 ± 111.86	**0.01**	**0.03**
Vitamin B1 (mg/day)^a,b^	1.96 ± 0.61	2.01 ± 0.69	2.29 ± 0.59	**0.001**	**<0.001**
Vitamin B2 (mg/day)^a,b^	2.00 ± 0.77	2.16 ± 0.89	2.42 ± 0.69	**0.001**	**<0.001**
Vitamin B3 (mg/day)^a,b^	23.46 ± 7.94	25.08 ± 11.76	27.31 ± 6.32	**0.01**	**0.003**
Pantothenic acid (mg/day)^b^	6.04 ± 2.02	6.25 ± 2.84	7.17 ± 2.02	**0.002**	**0.01**
Vitamin B6 (mg/day)^b^	2.02 ± 0.67	2.05 ± 0.76	2.42 ± 0.59	**<0.001**	**<0.001**
Vitamin B8 (mg/day)^a,b^	33.64 ± 14.00	37.47 ± 20.42	44.09 ± 13.42	**<0.001**	**<0.001**
Vitamin B9 (μg/day)^a,b,c^	558.34 ± 163.88	585.67 ± 177.49	679.13 ± 163.60	**<0.001**	**<0.001**
Vitamin B12 (μg/day)	4.36 ± 2.35	4.25 ± 2.53	4.36 ± 2.30	0.93	0.43

(–), not calculated; CHO, carbohydrates; DHA, docosahexaenoic acid; EPA, eicosapentaenoic acid; MUFA, monounsaturated fatty acids; N, number; PUFA, polyunsaturated fatty acids; SD, standard deviation; T, tertile.

* The *P*-values were obtained by the use of ANOVA.

** The *P*-values were obtained by the use of ANCOVA after adjustment for energy intake.

*P* < 0.05 was considered statistically significant and *P* = 0.05–0.07 was considered marginally significant.

Quantitative variables were obtained from one-way ANOVA and presented as mean ± SD.

Carbohydrate quality index includes total fiber, glycemic index, whole grains to total grains ratio, and solid carbohydrate to total carbohydrate ratio.

a Significant difference with Bonferroni analysis was seen between T1 and T2.

b Significant difference with Bonferroni analysis was seen between T1 and T3.

c Significant difference with Bonferroni analysis was seen between T2 and T3.

Bold values indicate significant and marginally significant *p*-values.

### CVD risk factors, anthropometric measures, and body composition of study participants among tertiles of the CQI

In the crude model, HOMA-IR (*P* = 0.003) and BF (*P* = 0.01) were significantly lower in participants with higher adherence to a high CQI diet, and DBP (*P* = 0.06) and BFM (*P* = 0.06) are marginally significantly lower in participants with higher adherence to a diet with high CQI. However, after adjusting for age, BMI, energy intake, and physical activity, HOMA-IR (*P* = 0.007), WC (*P* = 0.02), WHR (*P* = 0.007), BFM (*P* = 0.02), BF (*P* = 0.01), fat mass index (FMI) (*P* = 0.01), trunk fat (TF) (kg) (*P* = 0.01), and TF (%) (*P* = 0.009) were significantly lower and BMI (*P* = 0.06) is marginally significantly lower in participants with consumption of high CQI diet. There was no significant mean difference between the other CVD risk factors of participants across tertiles of the CQI, as shown in Table 4.

**Table 4:** CVD risk factors, anthropometric measures, and body composition of study participants among tertiles of the CQI (*n* = 291).

**CVD risk factors**	**Models**	**Tertiles of CQI**			*P*-value^*^	*P*-value^**^
		**T1**	**T2**	**T3**		
		***n* = 103**	***n* = 99**	***n* = 89**		
		**Mean ±SD**	**Mean ±SD**	**Mean ±SD**		
		**<10**	**10–13**	**>13**		
**Blood pressure**						
SBP (mmHg)	Crude	113.87 ± 12.90^1^	109.32 ± 13.58	111.63 ± 14.59	0.07	0.15
	Model 1	113.87 ± 1.29^2^	109.32 ± 1.40	111.63 ± 1.56		
DBP (mmHg)	Crude	79.60 ± 9.65	76.78 ± 9.29	76.74 ± 9.72	**0.06**	0.09
	Model 1	79.60 ± 0.97	76.78 ± 0.95	76.74 ± 1.04		
**Blood parameters**						
FBS (mg/dl)	Crude	87.57 ± 10.58	88.56 ± 9.93	86.26 ± 8.05	0.31	0.46
	Model 1	87.57 ± 1.14	88.56 ± 1.09	86.26 ± 0.91		
TC (mg/dl)	Crude	183.96 ± 35.08	188.42 ± 40.78	182.96 ± 32.39	0.59	0.93
	Model 1	183.96 ± 3.80	188.42 ± 4.47	182.96 ± 3.66		
TG (mg/dl)	Crude	115.27 ± 57.53	124.52 ± 63.67	114.97 ± 58.14	0.51	0.52
	Model 1	115.27 ± 6.24	124.52 ± 7.03	114.97 ± 6.66		
HDL (mg/dl)	Crude	46.44 ± 9.20	46.50 ± 11.99	47.52 ± 11.32	0.78	0.79
	Model 1	46.44 ± 0.99	46.50 ± 1.31	47.52 ± 1.28		
LDL (mg/dl)	Crude	95.60 ± 22.23	94.18 ± 26.93	95.32 ± 23.44	0.92	0.45
	Model 1	95.60 ± 2.41	94.18 ± 2.95	95.32 ± 2.65		
hs-CRP (mg/L)	Crude	4.94 ± 4.91	3.89 ± 4.57	4.12 ± 4.44	0.32	0.66
	Model 1	4.94 ± 0.55	3.89 ± 0.50	4.12 ± 0.51		
**Anthropometric parameters**						
NC (cm)	Crude	37.12 ± 2.61	37.37 ± 3.93	38.22 ± 12.08	0.65	0.60
	Model 1	37.12 ± 0.30	37.37 ± 0.48	38.22 ± 1.46		
WC (cm)^b^	Crude	100.26 ± 10.99	99.14 ± 9.94	97.26 ± 8.78	0.11	**0.02**
	Model 1	100.26 ± 1.08	99.14 ± 1.00	97.26 ± 0.93		
WHR^b^	Crude	0.93 ± 0.05	1.86 ± 9.19	0.92 ± 0.05	0.37	**0.007**
	Model 1	0.93 ± 0.00	1.86 ± 0.92	0.92 ± 0.005		
BMI (kg/m^2^)^b^	Crude	31.62 ± 4.96	31.15 ± 4.34	30.28 ± 3.33	0.09	**0.06**
	Model 1	31.62 ± 0.48	31.15 ± 0.43	30.28 ± 0.35		
**Body composition**						
BFM (kg)^b^	Crude	35.19 ± 9.91	34.32 ± 8.55	32.30 ± 6.91	**0.06**	**0.02**
	Model 1	35.19 ± 0.97	34.32 ± 0.86	32.30 ± 0.73		
FFM (kg)	Crude	47.23 ± 5.74	46.13 ± 5.38	46.97 ± 5.59	0.35	0.87
	Model 1	47.23 ± 0.56	46.13 ± 0.54	46.97 ± 0.59		
BF (%)^b^	Crude	42.06 ± 5.40	42.19 ± 5.24	40.13 ± 5.80	**0.01**	**0.01**
	Model 1	42.06 ± 0.53	42.19 ± 0.52	40.13 ± 0.61		
VFA (cm^2^)	Crude	165.94 ± 43.04	182.00 ± 171.14	155.88 ± 35.87	0.22	0.14
	Model 1	165.94 ± 4.24	182.00 ± 17.28	155.88 ± 3.80		
VFL (cm)	Crude	17.68 ± 19.34	15.70 ± 3.31	16.55 ± 14.59	0.61	0.74
	Model 1	17.68 ± 1.91	15.70 ± 0.33	16.55 ± 1.54		
FFMI	Crude	18.07 ± 1.61	17.82 ± 1.47	19.42 ± 13.90	0.33	0.99
	Model 1	18.07 ± 0.15	17.82 ± 0.14	19.42 ± 1.48		
FMI^b^	Crude	13.53 ± 3.73	13.41 ± 3.46	12.43 ± 2.76	**0.05**	**0.01**
	Model 1	13.53 ± 0.36	13.41 ±.034	12.43 ± 0.29		
TF (kg)^b^	Crude	17.01 ± 3.97	16.66 ± 3.62	15.94 ± 3.42	0.12	**0.01**
	Model 1	17.01 ± 0.39	16.66 ± 0.36	15.94 ± 0.36		
TF (%)^b^	Crude	322.09 ± 73.76	317.46 ± 72.24	299.98 ± 61.26	0.07	**0.009**
	Model 1	322.09 ± 7.26	317.46 ± 7.29	299.98 ± 6.49		
HOMA-IR index^a^	Crude	3.66 ± 1.50	3.38 ± 1.23	2.97 ± 0.97	**0.003**	**0.007**
	Model 1	3.66 ± 0.16	3.38 ± 0.14	2.97 ± 0.11		

BF, body fat percentage; BFM, body fat mass; BMI, body mass index; CQI, carbohydrate quality index; DBP, diastolic blood pressure; FBS, fasting blood sugar; FFM, fat-free mass; FFMI, fat-free mass index; FMI, fat mass index; HDL, high-density lipoprotein; HOMA-IR, homeostatic model assessment for insulin resistance; hs-CRP, high-sensitivity C-reactive protein; LDL, low-density lipoprotein; NC, neck circumference; SBP, systolic blood pressure; T, tertile; TC, total cholesterol; TF, trunk fat; TG, triglyceride; VFA, visceral fat area; VFL, visceral fat level; WC, waist circumference; WHR, waist-hip ratio.

Participants were divided into categories called tertiles.

* The *P-*values were obtained by the use of ANOVA.

** The *P*-values were obtained by the use of ANCOVA after adjustment for age, BMI, energy intake, and physical activity (MET minutes/week). BMI is considered a collinear variable for anthropometric ad body composition measurements.

*P* < 0.05 was considered statistically significant and *P* = 0.05–0.07 was considered marginally significant.

1 Unadjusted, mean ± SD.

2 Adjusted for Carbohydrate quality index includes total fiber, glycemic index, whole grains to total grains ratio, and solid carbohydrate to total carbohydrate ratio.

a Significant difference with Bonferroni analysis was seen between T1 and T3.

b Significant difference with Bonferroni analysis was seen between T2 and T3.

Bold values indicate significant and marginally significant *p*-values.

### The association between CQI with CVD risk factors, anthropometric measures, and body composition of study participants

According to Table 5, in the crude model, SBP (*P* = 0.02) and DBP (*P* = 0.04) had an inverse significant association with CQI in the second tertile. Furthermore, DBP (*P* = 0.04), HOMA-IR (*P* = 0.001), WC (*P* = 0.03), BMI (*P* = 0.03), BFM (*P* = 0.02), BF (*P* = 0.01), FMI (*P* = 0.02), TF (kg) (*P* = 0.04), and TF (%) (*P* = 0.02) had an inverse significant association with CQI in third tertile. After adjusting for confounding variables, such as age, BMI, energy intake, and physical activity, SBP (β = −6.10; 95% CI = −10.11, −2.10; *P* = 0.003) and DBP (β = −3.76; 95% CI = −6.63, −0.89; *P* = 0.01) had an inverse significant association with CQI in the second tertile. Furthermore, DBP (β = −3.11; 95% CI = −6.15, −0.08; *P* = 0.04), HOMA-IR (β = −0.53; 95% CI = −0.94, −0.12; *P* = 0.01), WC (β = −3.18; 95% CI = −6.26, −0.10; *P* = 0.04), WHR (β = −0.01; 95% CI = −0.03, −0.001; *P* = 0.03), BFM (β = −2.87; 95% CI = −5.48, −0.26; *P* = 0.03), BF (β = −2.06; 95% CI = −3.82, −0.30; *P* = 0.02), FMI (β = −1.07; 95% CI = −2.09, −0.04; *P* = 0.04), TF (kg) (β = −1.14; 95% CI = −2.28, −0.003; *P* = 0.04), and TF (%) (β = −22.98; 95% CI = −44.38, −1.57; *P* = 0.03) had an inverse significant association and BMI (β = −1.21; 95% CI = −2.50, 0.07; *P* = 0.06) had an inverse marginally significant association with CQI in the third tertile compare to T1. Based on this table, in crude model, more adherence to a higher CQI diet compare to lower adherence, was negatively significantly associated DBP (*P* for trend = 0.03), HOMA-IR (*P* for trend = 0.001), WC (*P* for trend = 0.03), BMI (*P* for trend = 0.03), BFM (*P* for trend = 0.02), BF (*P* for trend = 0.01), FMI (*P* for trend = 0.02), TF (kg) (*P* for trend = 0.04), and TF (%) (*P* for trend = 0.03). After adjusting for confounders, in model 1, greater adherence to a diet with high CQI was positively and significantly associated with a DBP (*P* for trend = 0.04), and negatively with HOMA-IR (*P* for trend = 0.01), WC (*P* for trend = 0.04), WHR (*P* for trend = 0.03), BFM (*P* for trend = 0.03), BF (*P* for trend = 0.02), FMI (*P* for trend = 0.04), and TF (%) (*P* for trend = 0.03), compare to reference group. Also, after adjusting for confounders, greater adherence to a higher CQI diet was positively and marginally significantly associated with SBP (*P* for trend = 0.05), and negatively with BMI (*P* for trend = 0.06) and TF (kg) (*P* for trend = 0.05).

**Table 5:** The association between CQI with CVD risk factors, anthropometric measures, and body composition of study participants (*n* = 291).

**CVD risk factors**	**Models**	**Tertiles**	**β ± SE**	**95% CI**	*P*-value^*^	***P* trend**
**Blood pressure**						
SBP (mmHg)	Crude	T2	−4.54 ± 1.95	−8.38, −0.71	**0.02**	0.23
		T3	−2.24 ± 1.99	−6.16, 1.67	0.26	
	Model 1	T2	−6.10 ± 2.04	−10.11, −2.10	**0.003**	**0.05**
		T3	−3.47 ± 2.15	−7.70, 0.75	0.10	
DBP (mmHg)	Crude	T2	−2.81 ± 1.36	−5.50, −0.13	**0.04**	**0.03**
		T3	−2.85 ± 1.39	−5.59, −0.12	**0.04**	
	Model 1	T2	−3.76 ± 1.46	−6.63, −0.89	**0.01**	**0.04**
		T3	−3.11 ± 1.54	−6.15, −0.08	**0.04**	
**Blood parameters**						
FBS (mg/dl)	Crude	T2	0.99 ± 1.47	−1.90, 3.88	0.50	0.40
		T3	−1.30 ± 1.49	−4.24, 1.63	0.38	
	Model 1	T2	1.07 ± 1.54	−1.94, 4.09	0.48	0.18
		T3	−2.14 ± 1.58	−5.25, 0.97	0.07	
TC (mg/dl)	Crude	T2	4.45 ± 5.57	−6.46, 15.37	0.42	0.88
		T3	−1.00 ± 5.66	−12.09, 10.09	0.85	
	Model 1	T2	5.50 ± 5.80	−5.87, 16.88	0.34	0.79
		T3	−1.58 ± 5.98	−13.30, 10.14	0.79	
TG (mg/dl)	Crude	T2	9.25 ± 9.20	−8.79, 27.30	0.31	0.99
		T3	−0.29 ± 9.39	−18.70, 18.11	0.97	
	Model 1	T2	16.15 ± 9.99	−3.43, 35.74	0.10	0.34
		T3	9.57 ± 10.34	−10.70, 29.86	0.35	
HDL (mg/dl)	Crude	T2	0.05 ± 1.66	−3.21, 3.33	0.97	0.53
		T3	1.07 ± 1.69	−2.24, 4.40	0.52	
	Model 1	T2	−0.71 ± 1.86	−4.36, 2.93	0.70	0.78
		T3	−0.52 ± 1.91	−4.28, 3.23	0.78	
LDL (mg/dl)	Crude	T2	−1.41 ± 3.72	−8.72, 5.88	0.70	0.93
		T3	−0.27 ± 3.78	−7.69, 7.13	0.94	
	Model 1	T2	−0.57 ± 3.98	−8.38, 7.22	0.88	0.85
		T3	−0.75 ± 4.10	−8.80, 7.28	0.85	
hs-CRP (mg/L)	Crude	T2	−1.05 ± 0.73	−2.48, 0.37	0.14	0.26
		T3	−0.82 ± 0.74	−2.28, 0.63	0.27	
	Model 1	T2	−0.13 ± 0.78	−1.67, 1.40	0.86	0.87
		T3	−0.13 ± 0.80	−1.70, 1.44	0.87	
**Anthropometric parameters**						
NC (cm)	Crude	T2	0.24 ± 1.24	−2.18, 2.68	0.84	0.37
		T3	1.10 ± 1.23	−1.31, 3.52	0.37	
	Model 1	T2	0.23 ± 1.47	−2.66, 3.12	0.87	0.38
		T3	1.28 ± 1.47	−1.61, 4.190	0.38	
WC (cm)	Crude	T2	−1.11 ± 1.40	−3.86, 1.63	0.42	**0.03**
		T3	−3.00 ± 1.44	−5.82, −0.17	**0.03**	
	Model 1	T2	−0.47 ± 1.50	−3.41, 2.46	0.75	**0.04**
		T3	−3.18 ± 1.57	−6.26, −0.10	**0.04**	
WHR	Crude	T2	0.92 ± 0.75	−0.54, 2.39	0.21	0.96
		T3	−0.01 ± 0.77	−1.52, 1.49	0.98	
	Model 1	T2	−0.001 ± 0.007	−0.01, 0.01	0.85	**0.03**
		T3	−0.01 ± 0.008	−0.03, −0.001	**0.03**	
BMI (kg/m^2^)	Crude	T2	−0.46 ± 0.60	−1.65, 0.71	0.43	**0.03**
		T3	−1.33 ± 0.62	−2.55, −0.12	**0.03**	
	Model 1	T2	−0.21 ± 0.62	−1.44, 1.01	0.73	**0.06**
		T3	−1.21 ± 0.65	−2.50, 0.07	**0.06**	
**Body composition**						
BFM (kg)	Crude	T2	−0.87 ± 1.21	−3.24, 1.49	0.46	**0.02**
		T3	−2.89 ± 1.24	−5.32, −0.46	**0.02**	
	Model 1	T2	−0.10 ± 1.27	−2.59, 2.38	0.93	**0.03**
		T3	−2.87 ± 1.33	−5.48, −0.26	**0.03**	
FFM (kg)	Crude	T2	−1.09 ± 0.78	−2.63, 0.43	0.16	0.70
		T3	−0.25 ± 0.80	−1.83, 1.31	0.75	
	Model 1	T2	−0.84 ± 0.87	−2.56, 0.87	0.33	0.92
		T3	0.09 ± 0.91	−1.70, 1.89	0.92	
BF (%)	Crude	T2	0.12 ± 0.76	−1.38, 1.63	0.87	**0.01**
		T3	−1.93 ± 0.78	−3.47, −0.38	**0.01**	
	Model 1	T2	0.53 ± 0.85	−1.15, 2.21	0.53	**0.02**
		T3	−2.06 ± 0.89	−3.82, −0.30	**0.02**	
VFA (cm^2^)	Crude	T2	16.06 ± 14.69	−12.73, 44.85	0.27	0.55
		T3	−10.06 ± 15.06	−39.59, 19.46	0.50	
	Model 1	T2	21.77 ± 18.30	−14.09, 57.63	0.23	0.53
		T3	−12.37 ± 19.15	−49.91, 25.16	0.51	
VFL (cm)	Crude	T2	−1.97 ± 1.99	−5.89, 1.93	0.32	0.56
		T3	−1.13 ± 2.04	−5.14, 2.88	0.58	
	Model 1	T2	−2.51 ± 2.53	−7.47, 2.45	0.32	0.51
		T3	−1.71 ± 2.64	−6.90, 3.48	0.51	
FFMI	Crude	T2	−0.24 ± 1.09	−2.38, 1.89	0.82	0.25
		T3	1.34 ± 1.12	−0.86, 3.54	0.23	
	Model 1	T2	−0.33 ± 1.35	−2.99, 2.33	0.80	0.28
		T3	1.54 ± 1.42	−1.24, 4.33	0.27	
FMI	Crude	T2	−0.12 ± 0.47	−1.04, 0.80	0.80	**0.02**
		T3	−1.10 ± 0.48	−2.05, −0.14	**0.02**	
	Model 1	T2	0.12 ± 0.49	−0.85, 1.09	0.81	**0.04**
		T3	−1.07 ± 0.52	−2.09, −0.04	**0.04**	
TF (kg)	Crude	T2	−0.34 ± 0.51	−1.36, 0.67	0.50	**0.04**
		T3	−1.07 ± 0.53	−2.11, −0.03	**0.04**	
	Model 1	T2	−0.11 ± 0.55	−1.20, 0.98	0.84	**0.05**
		T3	−1.14 ± 0.58	−2.28, −0.003	**0.04**	
TF (%)	Crude	T2	−4.63 ± 9.77	−23.78, 14.52	0.63	**0.03**
		T3	−22.10 ± 10.02	−41.75, −2.45	**0.02**	
	Model 1	T2	−1.29 ± 10.43	−21.74, 19.16	0.90	**0.03**
		T3	−22.98 ± 10.92	−44.38, −1.57	**0.03**	
HOMA-IR index	Crude	T2	−0.28 ± 0.19	−0.67, 0.10	0.15	**0.001**
		T3	−0.69 ± 0.20	−1.08, −0.29	**0.001**	
	Model 1	T2	−0.20 ± 0.20	−0.61, 0.19	0.31	**0.01**
		T3	−0.53 ± 0.21	−0.94, −0.12	**0.01**	

BF, body fat percentage; BFM, body fat mass; BMI, body mass index; CQI, carbohydrate quality index; DBP, diastolic blood pressure; FBS, fasting blood sugar; FFM, fat-free mass; FFMI, fat-free mass index; FMI, fat mass index; HDL, high-density lipoprotein; HOMA-IR, homeostatic model assessment for insulin resistance; hs-CRP, high-sensitivity C-reactive protein; LDL, low-density lipoprotein; NC, neck circumference; SBP, systolic blood pressure; TC, total cholesterol; TF, trunk fat; TG, triglyceride; VFA, visceral fat area; VFL, visceral fat level; WC, waist circumference; WHR, waist-hip ratio.

β and CI were obtained from linear regression and T1 is considered a reference group.

Participants were divided into categories called tertiles.

* The *P*-values were obtained by the use of linear regression after adjustment for age, BMI, energy intake, and physical activity.

*P* < 0.05 was considered statistically significant and *P* = 0.05–0.07 was considered marginally significant.

Carbohydrate quality index includes total fiber, glycemic index, whole grains to total grains ratio, and solid carbohydrate to total carbohydrate ratio.

Bold values indicate significant and marginally significant *p*-values.

## Discussion

To the best of our knowledge, the current study is the first study to investigate the association between CQI with CVD risk factors in Iranian women with obesity and overweight. The present study revealed after adjusting for age, BMI, energy intake, and physical activity, the consumption of a diet with high CQI was inversely related to blood pressure, insulin resistance, anthropometric measures, including WC, WHR, and BMI, and body composition, such as BF.

The results of a previous study showed a consistently inverse relationship between the CQI with the incidence of CVD. Indeed, these results emphasized that, in terms of the association between each of the CQI components with CVD, there was only a significant relationship between the whole grains/total grains ratio with CVD (33). Another study revealed an inverse association between CQI and CVD risk factors including HbA1c, FBS, TG, SBP, DBP, TC, and HDL (55). Fiber intake, as one of the CQI components, affects hypertension, metabolic syndrome components, insulin resistance, and LDL (54, 56–59). It also affects inflammatory markers such as hs-CRP (54, 56). GI, another CQI component, was shown to increase postprandial glucose, insulin responses, TG, and non-HDL cholesterol, and decrease HDL cholesterol (60, 61). Whole grains are one of the CQI components that in previous studies was shown to have significant effects on HDL, LDL, TC, HbA1c, and CRP (62). In the present study, an inverse significant association between SBP, DBP, and HOMA-IR with consumption of a high CQI diet was seen. Also, concordant with previous studies, an inverse relationship was seen between consumption of a diet with high CQI with LDL and hs-CRP, although their relationships were not significant (*P* > 0.05), which may be due to the small sample size. Even though the reduction in TC and FBS was observed with greater increases in CQI in previous studies (55), there was no association was seen between CQI with TC and FBS in our study. The small sample size may have contributed to this, therefore more studies with larger samples are needed.

In terms of anthropometric measures, some evidence has indicated a relationship between CQI with body weight and WC (55). A population-based study suggested an inverse association between CQI with abdominal obesity in men (23). Indeed, in previous studies, fiber intake was associated with obesity, WHR, WC, body weight, and BMI (54, 56, 63–65). Also, an association was seen between GI with body weight and obesity (66). In previous studies, an inverse association was seen between whole grains with central obesity and WC (67, 68). In addition, the results of a cohort study suggested a positive association between liquid carbohydrates with body weight (69). Concordant with these results, we concluded that there is a relationship between CQI with WHR, WC, and BMI. However, our results showed no significant association between CQI with body weight and abdominal or general obesity.

It has been revealed that an association exists between dietary fiber intake with skeletal muscle mass, BFM, and muscle-to-fat ratio (MFR) among women with type 2 diabetes (70), although one study showed no association (71). It has been asserted that diets rich in fiber can elicit weight-loss and BF loss compared to a diet high in refined grains (24). Also, low GI diet has been reported to cause BFM loss (26). In this study, we observed a strong relationship between consumption of a high CQI diet with body composition including BFM, BF, FMI, and TF.

Some possible mechanisms have been suggested pertaining to the association between CQI with CVD risk factors. Foods reach in fiber take more time to chew and so affect hunger reduction, increasing satiety, glucose control, improving insulin sensitivity, lipid absorption, lipid and carbohydrate oxidation regulation, and slowing down intestinal transit, that can cause body weight regulation (38, 39, 72, 73). Furthermore, fiber can fermentate in the colon by microflora and produce short-chain fatty acids (SCFAs), which contribute to improving health (74). Although the role of soluble and insoluble fiber is different, both are involved in reducing CVD risk factors and healthy body composition. Indeed, soluble fiber, due to its higher viscosity, induces satiety and controls hypercholesterolemia, and insoluble fiber, through inducing more satiety, and decreasing weight and WC, plays an important role (75–77). The mechanism by which dietary fiber lowers blood pressure levels remains unclear (78). Fermentation of dietary fiber in the intestine produces SCFAs. It has been seen that these SCFAs can lower blood pressure. The important mechanism through which SCFAs affect blood pressure is that SCFAs activate G protein-coupled receptors 43 and olfactory receptor 78 expressed in the kidney. This process inhibits the release of renin, which contributes to the regulation of blood pressure (79–81). Moreover, a high GI diet leads to insulin resistance, oxidative stress, and inflammation that aggravates dyslipidemia (82, 83). A high GI diet reduces fat oxidation and increases carbohydrate oxidation causing high-fat storage (84). On the other hand, a low GI diet leads to greater satiety and decreased desire for food intake, affecting energy intake and body composition balance (37). A high GI diet through increased postprandial insulin, causes activation of the sympathetic nervous system, sodium retention, and increased blood volume, resulting in increased blood pressure (85). Whole grains, as compared to refined grains, cause slower digestion and absorption of starch, and thus reduce insulin response and blood glucose. Also, whole grains induce greater satiety and reduce appetite leading to lower energy consumption and obesity prevention or improvement (38, 45). In addition, whole grains have been suggested to dilate blood vessels through the activity of endothelial nitric oxide, increase nitric oxide bioavailability, decrease inflammation, have antioxidant effects, increase arterial baroreceptor reflex function, and gut microbiota changes (86–89). So thus, decreasing blood pressure. Liquid carbohydrates, due to higher GI, increasing the risk of obesity (90); in addition, they can induce appetite, increase postprandial glucose and decrease insulin sensitivity compared to solid carbohydrates (43, 91).

The present study has several strengths. Based on our knowledge, this is the first study investigating the relationship between CQI with CVD risk factors. Also, we conducted this study on obese and overweight Iranian women, allowing detailed insight into this population. Despite these strengths, the study was not without some limitations. First, the sample size was relatively small and was performed only on women. Second, due to the cross-sectional design of the study, the findings do not establish causality between CQI with CVD risk factors. Third, we used the FFQ for obtaining the usual dietary intake which is based on participants' memory, thus may have resulted in recall bias. Forth, some measurement errors may have occurred while measuring.

## Conclusion

Consistent with previous studies, we found that consumption of a high CQI diet was negatively associated with blood pressure, insulin resistance (HOMA-IR), anthropometric measures, including WC, WHR, BMI, and body composition, such as BF. Clearly, further studies are warranted to confirm the veracity of these results.

## Data availability statement

The original contributions presented in the study are included in the article/supplementary material, further inquiries can be directed to the corresponding authors.

## Ethics statement

The studies involving human participants were reviewed and approved by the Local Ethics Committee of the Tehran University of Medical Sciences. The patients/participants provided their written informed consent to participate in this study.

## Author contributions

This project was designed and implemented by DK, AM, and KM. Data were analyzed and interpreted by FS. The manuscript was created by DK, CC, and AM. KM and AM supervised the overall project. This manuscript was revised by CC and ED. All authors have read and approved the final manuscript.

## Funding

This study was funded by the Tehran University of Health Sciences (Grant ID: 1400-2-212-55002).

## Conflict of interest

The authors declare that the research was conducted in the absence of any commercial or financial relationships that could be construed as a potential conflict of interest.

## Publisher's note

All claims expressed in this article are solely those of the authors and do not necessarily represent those of their affiliated organizations, or those of the publisher, the editors and the reviewers. Any product that may be evaluated in this article, or claim that may be made by its manufacturer, is not guaranteed or endorsed by the publisher.

## Abbreviations

ANCOVA: Analysis of Covariance
ANOVA: Analysis of Variance
BF: body fat percentage
BFM: body fat mass
BMI: body mass index
CIs: confidence intervals
CHO: carbohydrates
CVDs: cardiovascular diseases
CQI: carbohydrate quality index
DBP: diastolic blood pressure
DHA: docosahexaenoic acid
EPA: eicosapentaenoic acid
FBS: fasting blood sugar
FFM: fat-free mass
FFMI: fat free mass index
FFQ: food frequency questionnaires
FMI: fat mass index
HC: hip circumference
HDL: high-density lipoprotein
HOMA-IR: homeostatic model assessment for insulin resistance
hs-CRP: high-sensitivity C-reactive protein
IPAQ: International Physical Activity Questionnaire
LCD: low carbohydrate diet
LDL: low-density lipoprotein
MUFA: monounsaturated fatty acids
N: number
NC: neck circumference
PA: physical activity
PUFA: polyunsaturated fatty acids
SBP: systolic blood pressure
SD: standard deviation
T: tertile
TC: total cholesterol
TG: triglyceride
TF: trunk fat
TUMS: Tehran University of Medical Sciences
VFA: visceral fat area
VFL: visceral fat level
WC: waist circumference
WHO: world health organization
WHR: waist-hip ratio.
